# Correlation of carotid corrected flow time and respirophasic variation in blood flow peak velocity with stroke volume variation in elderly patients under general anaesthesia

**DOI:** 10.1186/s12871-022-01792-5

**Published:** 2022-08-04

**Authors:** Yu Chen, Ziyou Liu, Jun Fang, Yanhu Xie, Min Zhang, Jia Yang

**Affiliations:** Department of Anaesthesiology, Division of Life Sciences and Medicine, The First Affiliated Hospital of USTC, University of Sciences and Technology of China, Hefei, 230001 Anhui China

**Keywords:** Carotid Doppler ultrasound, Corrected flow time, Respirophasic variation, Stroke volume variation

## Abstract

**Background:**

Accurate assessment of volume responsiveness in elderly patients is important as it may reduce the risk of post-operative complications and enhance surgical recovery. This study evaluated the utility of two Doppler ultrasound-derived parameters, the carotid corrected flow time (FTc) and respirophasic variation in carotid artery blood flow peak velocity (ΔVpeak), to predict volume responsiveness in elderly patients under general anaesthesia.

**Methods:**

A total of 97 elderly patients undergoing elective abdominal surgery under general anaesthesia were enrolled in this prospective observational study. After entering the operating room, all patients underwent radial artery puncture connected with a LiDCO device to measure stroke volume variation (SVV), and fluid therapy was performed after anaesthesia induction. Patients were classified as responders if SVV ≥ 13% before fluid challenge and nonresponders if SVV < 13%. The FTc, ΔVpeak, SVV and haemodynamic data were measured by ultrasound at baseline (T0) and before (T1) and after (T2) fluid challenge. The correlations between the Doppler ultrasound-derived parameters and SVV were analysed, and the receiver operating characteristic (ROC) curves was computed to characterize both FTc and ΔVpeak as measures of volume responsiveness in elderly patients.

**Results:**

Forty-one (42.3%) patients were fluid responders. Carotid FTc before fluid challenge was negatively correlated with SVV before fluid challenge (*r* = -0.77; *P* < 0.01), and ΔVpeak was positively correlated with SVV (*r* = 0.72; *P* < 0.01). FTc and ΔVpeak predicted SVV ≥ 13% after general anaesthesia in elderly patients, with areas under the receiver operating characteristic curves (AUROCs) of 0.811 [95% confidence interval (CI), 0.721–0.900; *P* < 0.001] and 0.781 (95% CI, 0.686–0.875; *P* < 0.001), respectively. The optimal cut-off values of FTc and ΔVpeak to predict SVV ≥ 13% were 340.74 ms (sensitivity of 76.8%; specificity of 80.5%) and 11.69% (sensitivity of 78.0%; specificity of 67.9%), respectively.

**Conclusions:**

There was a good correlation between carotid artery ultrasound parameters and SVV. FTc predicted fluid responsiveness better than ΔVpeak in elderly patients during general anaesthesia. Further study is needed before these parameters can be recommended for clinical application.

**Trial registration:**

www.chictr.org.cn(ChiCTR2000031193); registered 23 March 2020.

## Background

The perioperative management of elderly patients is challenging for anaesthesiologists due to the functional decline of various organ systems and comorbidities; therefore, fluid management is critical [1]. Fluid overload can cause complications such as pulmonary oedema, heart failure, and acute kidney injury, while insufficient fluid resuscitation can exacerbate organ ischaemia and hypoxia, resulting in a poor prognosis [2, 3]. Therefore, we need more accurate and appropriate indicators to determine a patient's ability to respond to intravenous fluids, which may reduce complications and promote rehabilitation, especially for elderly patients.

Stroke volume variation (SVV) is a dynamic indicator based on heart–lung interactions that is now widely used in fluid management [4]. However, SVV monitoring is invasive and is difficult to implement generally in clinical practice due to its high cost and the need for specialized equipment. Recently, many investigators have used ultrasound technologyto guide perioperative fluid management [5]. New ultrasound indices are carotid corrected flow time (FTc) and respirophasic variation in carotid artery blood flow peak velocity (ΔVpeak) [6, 7]. FTc and ΔVpeak can accurately assess volume responsiveness and are also highly valuable for hypotension assessment after anaesthesia [8]. However, their accuracy in elderly patients following induction of anaesthesia has not been determined, nor have they been compared with SVV. Accordingly, the aim of this study was to evaluate the ability of carotid FTc and ΔVpeak to predict SVV ≥ 13% during mechanical ventilation in elderly patients following induction of general anaesthesia.

## Methods

### Study population

The study protocol was approved by the Institutional Ethics Committee of The First Affiliated Hospital of University of Science and Technology of China ( NO.2021KY096) and registered in the Chinese Clinical Trial Register (www.chictr.org.cn; ChiCTR2000031193). All methods were carried out in accordance with Declaration of Helsinki. After written informed consent was obtained from the participants, 100 patients were assessed, and 97 patients were enrolled in this prospective observational study between May 2021 and November 2021. Elderly patients (age 65 or older) of either sex with American Society of Anesthesiologists physical status ( ASA PS) I-III, cardiac function class I-II, and body mass index (BMI) 18–30 kg/m2 who underwent elective gastrointestinal surgery under general anaesthesia after fasting for at least 6 to 8 h were recruited for this study. Exclusion criteria were as follows: systolic blood pressure (SBP) ≥ 180 mmHg or diastolic blood pressure (DBP) ≥ 110 mmHg, arrhythmia, heart valve disease, peripheral vascular disease, chronic kidney disease, cerebrovascular disease, carotid artery stenosis > 50%, anatomical variation, history of previous neck surgery or neck trauma, chronic obstructive pulmonary disease, severe anaemia or hypotension, severe hypotension after anaesthesia induction (SBP less than 90 mmHg or vasoactive drugs used more than 3 times), and allergy to vasoactive drugs and colloids involved in the study.

### Study procedures

Upon arrival to the operating room,electrocardiogram ( ECG), noninvasive blood pressure measurement, pulse oximetry, and bispectral index (BIS) recording were commenced. Intravenous infusion of lactated Ringer solution (Tushan Pharmaceutical, Anhui, China) at 4 mL/kg/h was done through the peripheral vein. Radial artery puncture was performed under local anaesthesia to invasively monitor blood pressure and connect the patient to the LiDCO (LiDCO, Ltd, London, UK) system to monitor SVV. Patients were placed in a supine position in a calm state, and baseline carotid FTc, ΔVpeak and haemodynamic data were measured before general anaesthesia (T0). General anaesthesia was induced with etomidate at 0.15–0.2 mg/kg and sufentanil at 0.4–0.5 µg/kg, and endotracheal intubation was facilitated by intravenous rocuronium at 0.8 mg/kg. The patients were ventilated using volume control with a tidal volume of 8 -10 ml/kg, respiratory rate 8–12 breaths/min, inspiratory-to-expiratory ratio 1:2, positive end-expiratory pressure 0 cmH2O, and maintenance of end-tidal carbon dioxide partial pressure at 35–45 mmHg. Anaesthesia was maintained with propofol, remifentanil, and sevoflurane to keep the BIS between 40 and 60. The SVV, carotid FTc, ΔVpeak and haemodynamic values were measured 5 min after tracheal intubation (T1). At this time, the patients were categorized according to their SVV value; SVV ≥ 13% was the responder group, and < 13% was the nonresponder group. After recording these data, the patients were administered a fluid challenge of 7 ml/kg ideal body weight of 6% hydroxyethyl starch 130/0.4 (Volulyte, Fresenius Kabi Pharmaceuticals, Beijing, China) within 30 min. Five minutes after completion of the fluid challenge, the same haemodynamic parameters were measured again (T2). Measurements at T2 were divided into three cases: 1. Patients with an unopened abdomen and no pneumoperitoneum could be measured directly. 2. In patients with an established pneumoperitoneum, the pneumoperitoneum was released in collaboration with the surgeon, and the data were measured 5 min later. 3. If the patient's abdomen had been opened, the surgeon paused the abdominal operation and covered the incision with gauze before measuring.

### Carotid ultrasonography

**Fig. 1:**
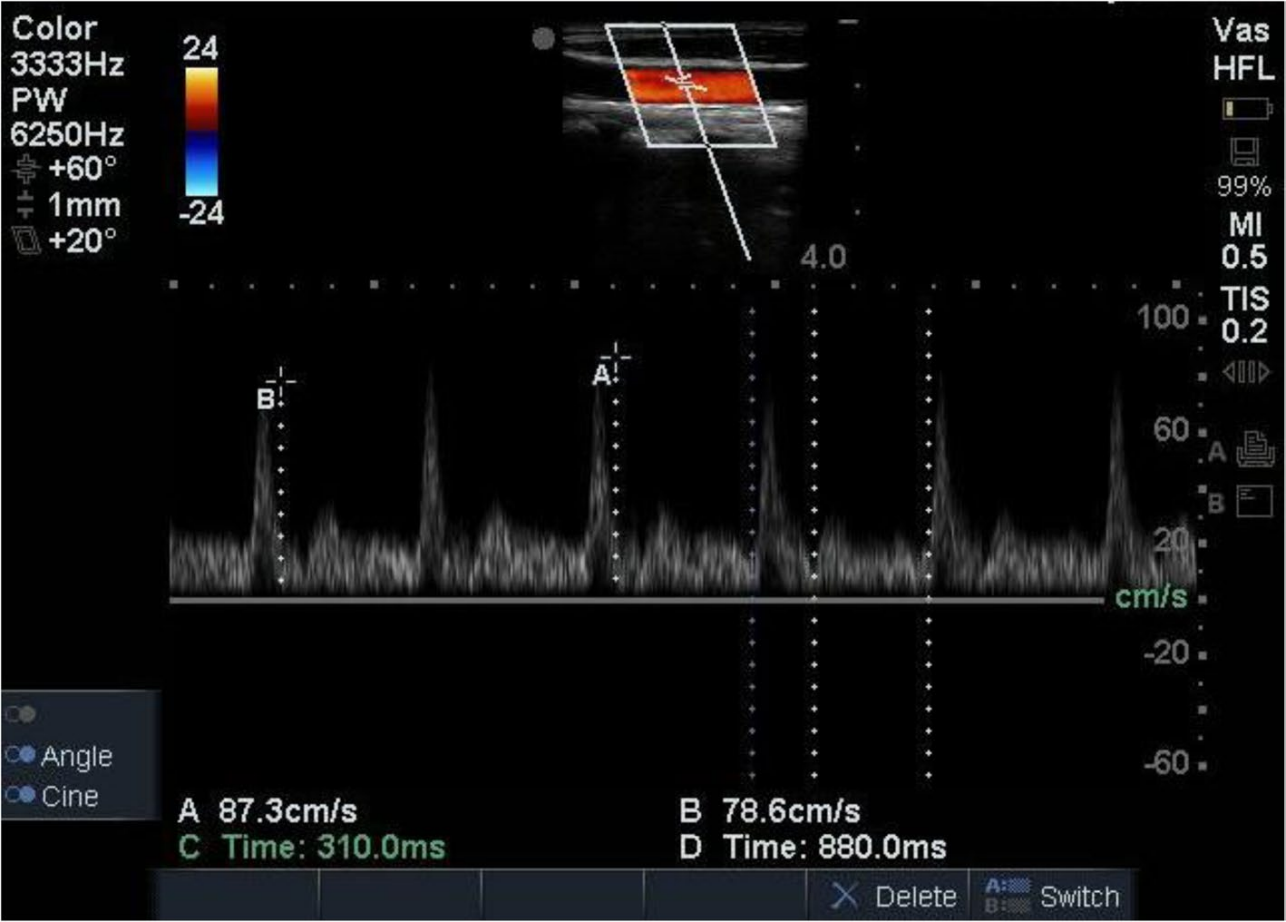
**C**arotid FTc and ΔVpeak measurement with Doppler ultrasound.Time C is FT.*FTc* carotid corrected flow time,*ΔVpeak* respirophasic variation in carotid artery blood flow peak velocity,*FT* flow time

FTc and ΔVpeak were measured by carotid ultrasound in the right common carotid artery as described by Blehar et al. [9] and Song et al. [7]. Carotid FTc and ΔVpeak were measured with a Sonosite ultrasound machine (SonoSite Fujifilm Edge, Bothell, WA, USA) by two independent examiners (Fig. 1). The examiners were blinded to each other's Doppler results and the patient's haemodynamic parameters. Patients were placed in a supine position with the head tilted to the left, and a 6–13.0-MHz linear array transducer was placed longitudinally on the neck with the probe marker pointing to the patient’s head. The long-axis B-mode image of the right common carotid artery was obtained at the level of the lower border of the thyroid cartilage. Then, the sample volume was placed on the centre of the lumen, approximately 2 cm proximal to the carotid bifurcation. Next, pulsed wave Doppler tracing of the flow through the artery was done with angle correction. After obtaining images that were stable and of acceptable quality, we saved the images and later measured its using the calliper function on the machine. The flow time (FT) was obtained by measuring the interval between the systolic upstroke and dicrotic notch. FTc was calculated using the simplified formula: FTc = FT + [1.29 × (heart rate—60)] [10–12]. Two examiners each performed the FTc measurements twice to generate four values; the mean of these values was used for analysis.

ΔVpeak was measured the same way FTc was. The maximum and minimum systolic velocity peaks were obtained in a single respiratory cycle. The calculation method ofΔVpeak was as follows: ΔVpeak = (maximum peak velocity-minimum peak velocity)/ [(maximum peak velocity + minimum peak velocity)/2] × 100. Each examiner measured it twice, and the mean of the four values was used for analysis.

### Statistics

Sample-size calculation was performed with the Tests for One ROC Curve module in PASS 11 software. Previous studies have reported that the area under the receiver operating characteristic (ROC) curve (AUROC) of the descending aortic FTc to predict fluid responsiveness was 0.82 [13], so we assumed that the AUROC of the carotid FTc was 0.7, which was quite low value. The sample size calculation estimated at least 82 patients were needed. To allow for a 10% dropout rate, 100 patients were recruited to this study.

Normality distribution was assessed using the Kolmogorov–Smirnov and Shapiro–Wilk tests. Data are presented as the mean and standard deviation for normally distributed continuous variables and as absolute numbers or percentages for categorical variables. Nonnormally distributed variables are reported as medians and interquartile ranges. Responder and nonresponder groups were compared by the independent t test for normally distributed data, the Mann–Whitney U test for nonnormally distributed data, and the χ^2^ test for categorical variables.

The Pearson correlation coefficient was calculated to assess the relationships between ultrasound parameters and SVV. ROC curve analysis was performed to assess the ability of the ultrasound-derived parameters FTc and ΔVpeak to predict SVV ≥ 13%. The best cut-off value was that which maximized the Youden index [14] and the confidence intervals for ROC analysis were calculated using the ‘pROC’ package within R. The grey area method described by Coste and Pouchot was used to determine the uncertainty range of the carotid measurements [15]. The cut-off values for FTc and ΔVpeak that had a sensitivity of 90% and specificity of 90% determined the boundaries of the grey zone. All statistical analyses were conducted with SPSS 19.0. A *P-value* < 0.05 (two-tailed) was considered statistically significant.

## Results

**Fig. 2:**
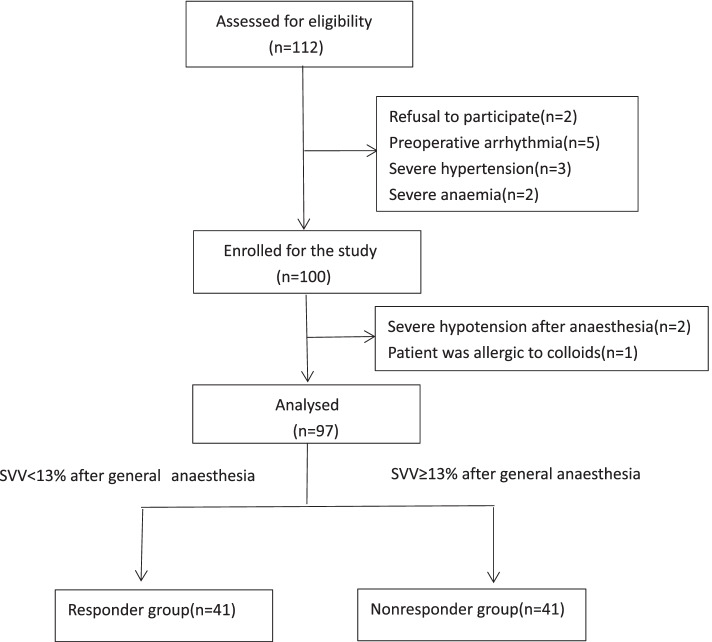
Flow diagram of patient enrolment.*SVV* stroke volume variation

A total of 112 eligible patients were evaluated, of whom 12 were excluded due to arrhythmia (*n* = 5), severe hypertension (*n* = 3), severe anaemia (*n* = 2), or refusal to participate (*n* = 2). One hundred subjects were eventually enrolled. During the study, 2 patients developed severe hypotension after anaesthesia, and 1 patient was allergic to colloids. After they were excluded, 97 patients were included in the final analysis (Fig. 2). Patient characteristics were comparable between responders (*n* = 41) and nonresponders (*n* = 56) (Table 1). The baseline SBP, DBP, mean arterial pressure (MAP) and heart rate (HR) were not different between the two groups (*P* = 0.42, *P* = 0.95, *P* = 0.66, *P* = 0.65). However, the common carotid FTc was significantly lower (*P* < 0.001) and the ΔVpeak was significantly higher (*P* < 0.001) in the responder group.

**Table 1:** Patient baseline characteristics and haemodynamic parameters

	**All patients** **(*n* = 97)**	**Responders (*n* = 41)**	**Nonresponders (*n* = 56)**	***P-*value**
Male/female	61/36	25/16	36/20	0.83^b^
Age, y	71.6(65–88)	71.2(65–84)	71.9(65–88)	0.50^c^
BMI, kg/m2	23.0 ± 2.5	23.2 ± 2.6	22.8 ± 2.5	0.37^a^
ASA PS I/II/III	16/52/29	6/20/15	10/32/14	0.47^b^
Diagnosis				
Stomach tumour	26	12	14	0.64^b^
Intestinal tumour	63	27	36	1.00^b^
Enterostomy closure	8	2	6	0.46^b^
Type of surgery				
Laparoscopic surgery	89	39	50	0.26^b^
Open surgery	8	2	6	0.26^b^
Comorbidities				
Hypertension	18	8	10	1.00^b^
Diabetes mellitus	7	3	4	1.00^b^
Coronary artery disease	3	2	1	0.57^b^
Baseline SBP, mmHg	130.9 ± 10.7	129.8 ± 12.0	131.6 ± 9.8	0.42^a^
Baseline DBP, mmHg	71.0 ± 8.1	71 ± 8.6	70.9 ± 7.8	0.95^a^
Baseline MAP, mmHg	90.9 ± 7.9	90.5 ± 7.8	91.2 ± 8.1	0.66^a^
Baseline HR, beats/min	69.2 ± 9.0	68.7 ± 10.1	69.6 ± 8.3	0.65^a^
Baseline carotid FTc, ms	343.2 ± 10.1	337.2 ± 9.1	347.6 ± 8.5	< 0.001^a^
Baseline ΔVpeak, %	10.7 ± 2.3	12.4 ± 1.8	9.5 ± 1.8	< 0.001^a^

Data are presented as the mean (standard deviation) or median (interquartile range)

^a^ Independent-sample t test

^b^ χ2 test

^c^ Mann–Whitney U test

*BMI* Body mass index, *ASA PS* American Society of Anesthesiologists physical status, *SBP* systolic blood pressure, *DBP* diastolic blood pressure, *MAP* mean arterial pressure, *HR* heart rate, *FTc* carotid corrected flow time, *ΔVpeak* respirophasic variation in carotid artery blood flow peak velocity

**Fig. 3:**
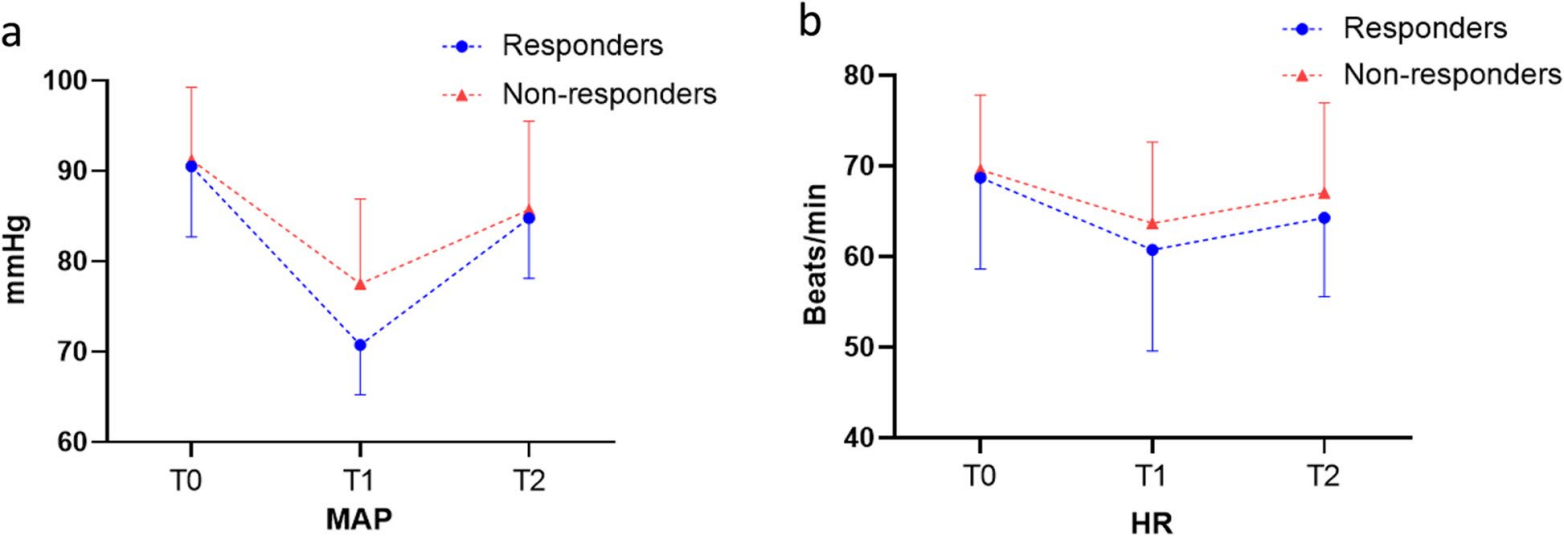
Haemodynamic data(MAP,HR) in two groups at T0,T1 and T2.*MAP* mean arterial pressure,*HR* heart rate, *T0* before general anaesthesia,*T1* 5 min after tracheal intubation,*T2* 5 min after completion of the fluid challenge

**Fig. 4:**
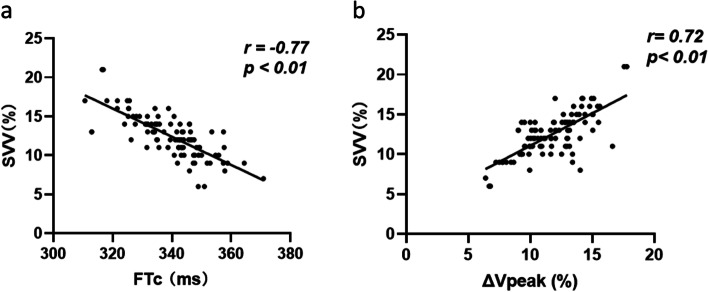
The relationship between SVV and FTc or ΔVpeak at T1.Trend lines are presented as solid lines.*SVV* stroke volume variation,*FTc* carotid corrected flow time,*ΔVpeak* respirophasic variation in carotid artery blood flow peak velocity,*T1* 5 min after tracheal intubation

The haemodynamic variables at T1 and T2 in the presence of mechanical ventilation are shown in Fig. 3 and Table 2. In the responders, MAP and HR fluctuated more, especially MAP. In both groups, fluid therapy significantly increased FTc and significantly decreased SVV and ΔVpeak. Before fluid therapy, FTc was significantly lower in responders than in nonresponders, and this was still true after the fluid challenge. In contrast, SVV and ΔVpeak were significantly higher in responders than in nonresponders before fluid replacement therapy, and ΔVpeak remained significantly higher in responders than in nonresponders after fluid replacement therapy. FTc and ΔVpeak during mechanical ventilation after general anaesthesia were associated with SVV at T1 (*r* = -0.77, *P* < 0.01; *r* = 0.72, *P* < 0.01, respectively; Fig. 4).

**Table 2:** Haemodynamic variables at T1 and T2

	**Responders**		**Nonresponders**	
	**T1**	**T2**	**T1**	**T2**
SVV (%)	14.7(13–21)	9.1(4–13) ^a^	10.5(6–14) ^b^	6.5(2–11) ^ab^
FTc (ms)	332.6 ± 9.9	356.4 ± 8.8^a^	344.2 ± 10.4^b^	363.6 ± 8.2^ab^
ΔVpeak (%)	13.1 ± 2.2	8.9 ± 1.5^a^	10.8 ± 2.2^b^	7.2 ± 1.8^ab^

Data are presented as the mean (standard deviation) or median (interquartile range)

^a^ *P* < 0.05 vs. T1

^b^ *P* < 0.05 vs. responders

*T1* 5 min after tracheal intubation,*T2* 5 min after completion of the fluid challenge, *SVV* stroke volume variation

**Fig. 5:**
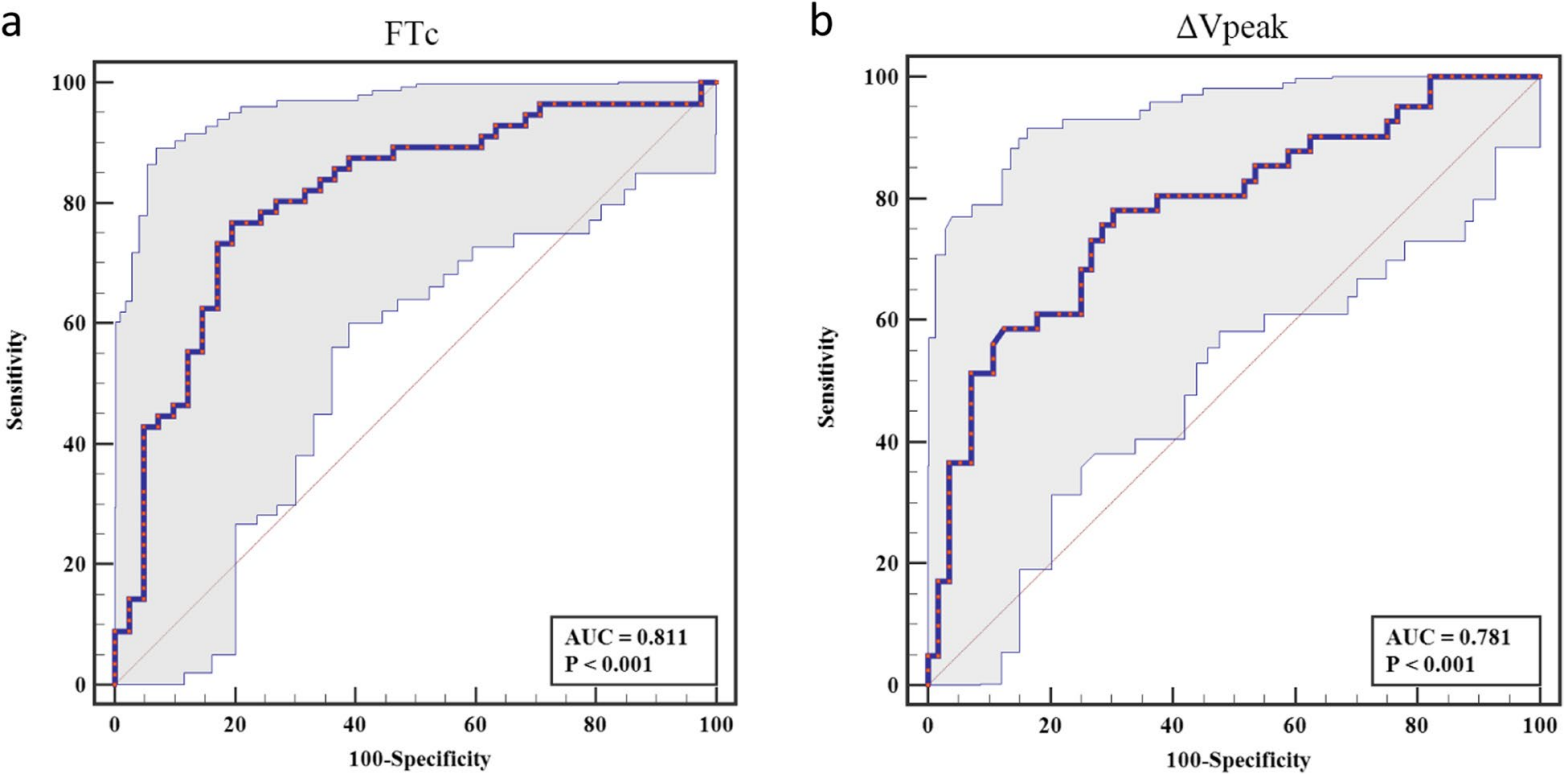
Area under the receiver operating characteristic curve of the carotid FTc and ΔVpeak to predict the ability of SVV ≥ 13%.Shaded area represents 95% confidence interval.*FTc* carotid corrected flow time,*ΔVpeak* respirophasic variation in carotid artery blood flow peak velocity,*SVV* stroke volume variation

The ability of FTc and ΔVpeak to predict SVV ≥ 13% at T1 is shown in Fig. 5 and Table 3. The AUROCs for FTc and ΔVpeak were 0.811 (95% confidence interval [CI], 0.721–0.900, *P* < 0.001) and 0.781 (95% CI, 0.686–0.875, *P* < 0.001), respectively. The best cut-off value of FTc obtained from this study was 340.74 ms, with a sensitivity of 76.8% and specificity of 80.5%. The optimal cut-off value of ΔVpeak was 11.69%, with a sensitivity of 78% and specificity of 67.9%.The grey zone for FTc occurred between 331.44 and 345.90 ms and contained 47.4% of patients. The grey zone for ΔVpeak occurred between 9.97% and 13.42% and included 48.5% of patients.

**Table 3:** Diagnostic performance of carotid FTc and ΔVpeak to predict the ability of SVV ≥ 13%

Parameter	AUROC (95% CI)	*P-value*	Best Cut-off	grey zone	Patients in grey zone(%)	Sensitivity (%)	Specificity (%)	Youden index
FTc	0.811(0.721–0.900)	< 0.01	340.74	331.44–345.90	47.4	76.8	80.5	0.57
ΔVpeak	0.781(0.686–0.875)	< 0.01	11.69	9.97–13.42	48.5	78	67.9	0.48

*AUROC* Area under the receiver operating characteristic curve, *FTc* Carotid corrected flow time, *ΔVpeak* Respirophasic variation in carotid artery blood flow peak velocity

## Discussion

This study showed that ultrasound assessment of carotid artery FTc was a more accurate and reliable indicator of volume responsiveness in elderly patients under general anaesthesia. ΔVpeak could also predict SVV ≥ 13% well. The changes in FTc and ΔVpeak from before to after fluid treatment were opposite: FTc increased, while ΔVpeak decreased.

The ability of carotid FTc to predict fluid responsiveness based on transoesophageal ultrasound measurement of descending aortic FTc has proven useful for volume optimization [9]. Kim et al. [16] reported that FTc could better predict fluid response in patients who breathed spontaneously, with an AUROC of 0.811. However, related studies did not analyse different populations, and the study population was largely young patients, so the findings might not apply to all populations. Elderly patients are likely to have chronic underlying diseases such as hypertension, diabetes, and arterial plaque causing decreased elasticity of blood vessels [17]. This study is the first to evaluate the ability of carotid FTc to predict fluid responsiveness in elderly patients during mechanical ventilation using carotid ultrasonography.

In previous studies of carotid ultrasound, various hemodynamic measures were used as the reference standards to determine volume responsiveness. These included invasive monitoring, MAP and noninvasive ultrasound monitoring [18]. Among them, it was more common to use the change in stroke volume (SV) or stroke volume index (SVI) after fluid therapy as a measure of volume responsiveness [19, 20]. However, we used SVV as the grouping criterion because SVV is currently considered the most reliable indicator of volume responsiveness and its most common diagnostic threshold is 13%, though SVV is reliable only under strictly controlled conditions (e.g., tidal volume ≥ 8 m l/kg ideal body weight and an absence of arrhythmias) [21–23]. Accordingly, the aim of our study was to indirectly demonstrate the ability of carotid artery Doppler ultrasonography to predict fluid responsiveness by assessing its relationship with SVV. The purpose of giving fluid therapy to all patients after grouping was to understand the trend of changes FTc and ΔVpeak. It was easier to use SVV in this study to evaluate and group the patients’ volume responsiveness to investigate the correlation between FTc, ΔVpeak and SVV. Given that we observed good correlations between carotid Doppler parameters and SVV, these ultrasound measures might be useful determinants of volume responsiveness, however, these conclusions are tempered by the relatively large grey zone analysis for both measures. That is, approximately 50% of the data for both FTc and ΔVpeak fell within the grey zone.

Curiously, FTc also increased more following fluid administration in the non-responsive group, which is inconsistent with the findings of Kimura A et al. [20] and Xu L et al. [24]. The reasons for this discrepancy may be the following: 1. Most of the medical records selected in this study were of laparoscopic abdominal surgery. These patients may be in a state of artificial pneumoperitoneum at time T2. Although we relieved the pneumoperitoneum with the cooperation of the surgeon and returned the patient to the supine position for measurement after 5 min, the influence of pneumoperitoneum on the patient may not have completely disappeared. 2. We grouped patients by the value of SVV. There are also certain errors in using SVV to measure fluid responsiveness. If the change in SV or SVI after fluid therapy were used as the criterion for volume responsiveness, there may be patients in the nonresponder group defined by SVV who are defined as responders. 3. In order to synchronize the ultrasound parameters and the LiDCO as much as possible, the time for each carotid ultrasound measurement was very short, which also increased the error of manual measurement.

ΔVpeak also well predicted SVV ≥ 13% in mechanically ventilated elderly subjects, with an AUROCof 0.781 and a cut-off value of 11.69%. Compared with Song and colleagues' study [7] of patients with mechanical ventilation with coronary artery disease and Kim's study [16] of patients with spontaneous breathing, this study found lower values for the predictability of ΔVpeak. This may be due to spontaneous breathing, which changes ventricular loading and/or diminished vascular elasticity in elderly patients. 

It is worth mentioning that the changes in haemodynamic parameters were different in the two groups. The FTc value of the responder group was lower than that of the nonresponder group, while the ΔVpeak value was significantly higher in the nonresponder group. After anaesthesia induction, the blood pressure and heart rate of the subjects in the two groups decreased, but the decrease in the responder group was pronounced. All of these results suggest that subjects in the responder group had volume deficits and were more prone to haemodynamic fluctuations due to the effects of anaesthetic drugs.

Our study has some limitations. First, the type of surgery was divided into laparoscopic surgery and open surgery. Although the measurement time for patients with pneumoperitoneum was 5 min after the pneumoperitoneum was relieved, the pneumoperitoneum may still have affected the measurement results [25, 26]. Second, FTc is a summative measure reflecting preload, afterload and contractility. Therefore, many other conditions that change FTc may have driven our observed differences between responders and non-responders [27]. Third, we used LiDCO to monitor SVV values, but this method may be less reliabe than measurement by PICCO monitors [28]. Fourth, although the population of this study was elderly patients, they all had acceptable cardiorespiratory function withwell-controlled and mild comorbidities. Further studies are needed to determine whether the results can be applied to all elderly patients, especially those with severe comorbidities. The correlation between FTc and SVV should be tested in different patient populations and clinical settings.

In conclusion, we found that the carotid FTc and ΔVpeak of elderly patients have good correlations with SVV, so they perform moderately well at predicting volume responsiveness. Considering its higher AUROC, as well as its slightly smaller grey area, FTc could be a more feasible and reliable predictor of SVV ≥ 13% than ΔVpeak. Further studies are needed to identify its role in other types of surgery and high-risk patients, such as those with severe cardiovascular disease.

## Data Availability

The datasets used and analysed during this study are available from the corresponding author on reasonable request.

## Abbreviations

FTc: Carotid corrected flow time
ΔVpeak: Respirophasic variation in carotid artery blood flow peak velocity
SVV: Stroke volume variation
ROC: Receiver operating characteristic
AUROC: Area under the receiver operating characteristic curve
ASA PS: American Society of Anesthesiologists physical status
BMI: Body mass index
SBP: Systolic blood pressure
DBP: Diastolic blood pressure
ECG: Electrocardiogram
BIS: Bispectral Index
FT: Flow time
MAP: Mean arterial pressure
HR: Heart rate
SV: Stroke volume
SVI: Stroke volume index
