# Correction: Correlation of carotid corrected flow time and respirophasic variation in blood flow peak velocity with stroke volume variation in elderly patients under general anaesthesi

**DOI:** 10.1186/s12871-023-02058-4

**Published:** 2023-04-13

**Authors:** Yu Chen, Ziyou Liu, Jun Fang, Yanhu Xie, Min Zhang, Jia Yang

**Affiliations:** Department of Anaesthesiology, The First Affiliated Hospital of USTC, Division of Life Sciences and Medicine, University of Science and Technology of China, Hefei, Anhui 230001 China

**Correction: BMC Anesthesiol 22, 246 (2022)** 10.1186/s12871-022-01792-5

Following publication of the original article [1], the authors reported an error to their affiliation. The correct should be “Department of Anaesthesiology, The First Affiliated Hospital of USTC, Division of Life Sciences and Medicine, University of Science and Technology of China, Hefei, Anhui 230001, China”.

The original article [1] has been updated.
